# Optogenetic interrogation of cell signalling: human neuropsin (hOPN5) represents a potent tool for controlling the Gq pathway with light

**DOI:** 10.1007/s00424-022-02765-w

**Published:** 2022-11-02

**Authors:** Siri Leemann, Sonja Kleinlogel, Franziska Schneider-Warme

**Affiliations:** 1 Institute for Experimental Cardiovascular Medicine, University Heart Center Freiburg - Bad Krozingen, Faculty of Medicine, University of Freiburg, Freiburg, Germany; 2 Institute of Physiology, Department of Biomedical Research (DBMR), University of Bern, Bern, Switzerland

G protein coupled receptors (GPCR) are ubiquitously expressed cell-surface receptors involved in the regulation of most physiological processes. This universality makes GPCR extremely attractive as both research tools and pharmacological targets. Upon stimulation of a particular GPCR, distinct intracellular signalling pathways are activated via heterotrimeric G proteins, themselves divided into four major families, Gi/o, Gq/11, Gs and G12/13.

GPCR signalling is classically modulated by pharmacological agents, lacking spatial and temporal resolution. This prevents the study of cell-type- and compartment-specific signalling on physiologically relevant timescales. Optogenetics can overcome these limitations by combining cell-type-specific genetic targeting with spatiotemporally precise optical stimulation. The first optogenetic studies were performed in 1988 by Khorana and colleagues that heterologously expressed bovine rhodopsin in *Xenopus* oocytes to elicit light-induced inward currents [4]. In 2003, Zemelman et al. optically triggered action potentials in vertebrate neurons co-expressing *Drosophila* arrestin-2, rhodopsin and the α-subunit of the cognate G protein [10]. However, applications of optogenetically controlled GPCR (opto-GPCR) remained scarce [3, 5], probably due to the complexity of intracellular signalling, difficulty of exogenous expression and the concurrent rise of microbial one-component optogenetics.

Ideally, opto-GPCR should be non-promiscuous and specific for only one G protein signalling pathway. Further, they should be easily switched between G protein activated and deactivated states and effectively drive downstream signalling at low, non-phototoxic light levels. Recently, opto-GPCR optogenetics was fostered by the identification of novel rhodopsins across different vertebrate and invertebrate species as well as the increasing availability of high-resolution GPCR structures in the active and inactive states.

One interesting candidate for optogenetic applications is the bistable mammalian neuropsin (OPN5) [7, 9] found in neuronal tissue and shown to be involved in photoentrainment and thermogenesis. In a recent ground-breaking study published in *Nature Communications*, Wagdi et al. elegantly showed that human OPN5 (hOPN5) signals specifically through Gq [8] and lacks promiscuity as found in most other Gq-coupled opto-GPCR, including melanopsin [1, 6]. In a separate study, Dai et al. controlled Gq signalling with the chicken orthologue of OPN5 [2], confirming OPN5’s utility as a potent optogenetic tool.

To dissect hOPN5 signalling, Wagdi et al. first expressed hOPN5 in HEK cells and determined Gq signalling by IP1 levels, a degradation product of IP3, as well as by semi-quantitative Ca^2+^ imaging (Fig. 1). The absence of Gi/o signalling found in most other Gq-coupled opto-GPCR was convincingly shown in HEK293-GIRK cells expressing a G protein coupled inwardly-rectifying potassium channel (GIRK) modulated by both, Gi/o and Gq. The authors then assessed optogenetic control of beating rate and contractility in diverse muscle cells. Light stimulation of embryonic stem cell-derived cardiomyocytes and of intact mouse hearts expressing hOPN5 equally led to positive chronotropic effects, which were completely abolished by application of a specific Gq blocker. hOPN5 activation also caused positive inotropic effects in murine adult cardiomyocytes and directly induced Gq-mediated contractions in small intestine, bladder and uterine smooth muscle cells. Finally, hOPN5 stimulation was employed in a biotechnological application by establishing an all-optical high-throughput screening (HTS) assay on Gq-coupled TRPC6 channels, an important drug target since involved in glomerulosclerosis and pulmonary hypertension. All-optical HTS with hOPN5 turned out to be much more specific and more sensitive than conventional HTS methods.

**Fig. 1:**
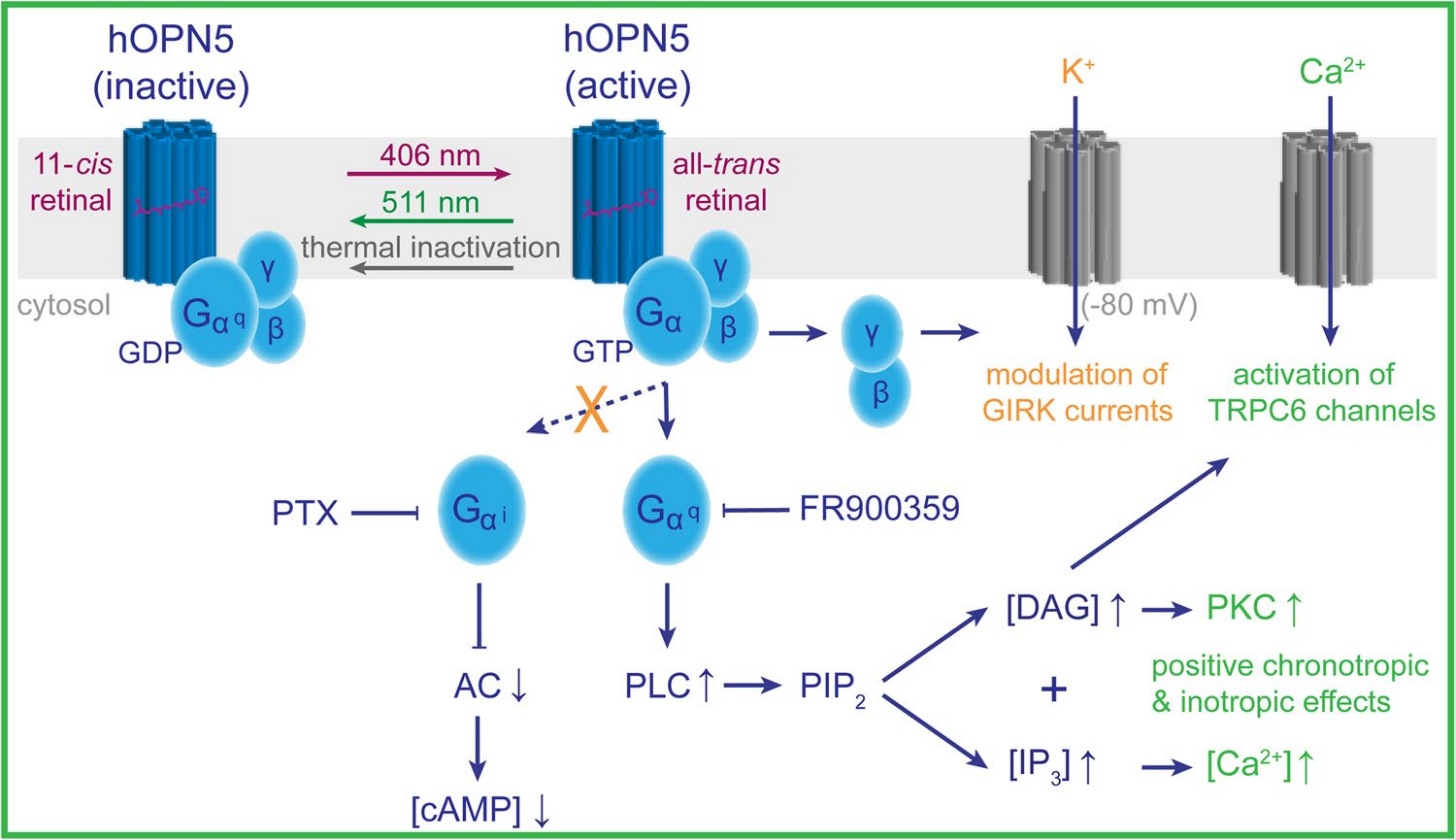
hOPN5-mediated activation of Gq signalling pathways as suggested by Wagdi and colleagues [8]. Human OPN5, a seven-transmembrane opto-GPCR, is activated by near-ultraviolet light (406 nm) and deactivated by green light (511 nm). Upon activation, hOPN5 exclusively triggers the Gq downstream signalling pathway. The Gαq subunit of the heterotrimeric G protein acts via phospholipase C (PLC) catalysing the conversion of phosphatidylinositol 4,5-bisphosphate (PIP_2_) into diacylglycerol (DAG) and inositol 1,4,5-trisphosphate (IP_3_). In turn, DAG activates protein kinase C (PKC) and canonical transient receptor potential (TRPC) channels. IP_3_ triggers the release of Ca^2+^ from intracellular stores, thereby inducing positive chronotropic (increase in rate) and inotropic (increase in contractility) responses in muscle cells. Based on a G protein coupled inwardly-rectifying potassium channel (GIRK) assay combined with pharmacological inhibition of either Gq or Gi proteins using FR900359 or pertussis toxin (PTX), respectively, the authors excluded the activation of Gi signalling pathways, which would decrease the intracellular cAMP levels by inhibiting adenylyl cyclase (AC) and would increase GIRK currents. In contrary, GIRK currents were reduced due to PLC-dependent PIP_2_ depletion

Using an opto-GPCR that is commonly expressed in human tissue provides a favourable opportunity for translational approaches as its immunogenicity is minimal. Although hOPN5 was robustly activated at low light levels of only 1 µW/mm^2^, enabling applications in intact tissues and organs, the development of red-shifted hOPN5-based receptors could improve non-invasive deep tissue stimulation. Albeit not further exploited, hOPN5 was also shown to be bi-stable with excellently separated activation (406 nm) and deactivation spectra (511 nm). In summary, the study by Wagdi et al. gives clear evidence for hOPN5’s Gq-specificity and adds exciting prospects for opto-GPCR applications in vitro, in drug screening assays, and potentially even in vivo.

## Data Availability

Not applicable.
